# Machine Learning-Accelerated Analysis of *In Utero* Embryo Phenotyping in *C. elegans* for Reproductive Toxicity Assessment

**DOI:** 10.21203/rs.3.rs-9986153/v1

**Published:** 2026-08-03

**Authors:** Abhishri Medewar, Andrew DuPlissis, Adam Laing, Amber Shen, Evan Hegarty, Sebastian Gomez, Gina Carrion, Julia Brown, Sudip Mondal, Adela Ben-Yakar

**Affiliations:** 1 vivoVerse, LLC, Austin, TX 78731, USA; 2 Department of Mechanical Engineering, The University of Texas at Austin, TX 78712, USA; 3 Department of Biomedical Engineering, The University of Texas at Austin, TX 78712, USA

**Keywords:** Machine learning, artificial intelligence, developmental and reproductive toxicity (DART), *C. elegans*, *in utero* embryo phenotype, high-resolution imaging, microfluidic technology, repeatable assays, acceptance criteria, automated analysis

## Abstract

Predictive new approach methodologies (NAMs) for developmental and reproductive toxicity (DART) assessment are increasingly needed as reliance on conventional mammalian studies decreases and chemical safety evaluation demands continue to expand. Whole-organism NAMs, including *Caenorhabditis elegans*, provide a scalable non-mammalian strategy because they preserve conserved biological pathways within an intact physiological system. We recently developed vivoDART, a rapid and repeatable *C. elegans* assay that quantifies *in utero* embryo development and overcomes key limitations of traditional labor-intensive, multiday *C. elegans* DART workflows. However, despite its robustness and reproducibility, vivoDART still requires manual analysis of tens of thousands of embryos per chemical, a process that is time-consuming and prone to user-dependent variability. To address this bottleneck, we developed EmbryoMAE-Det, a machine-learning framework trained on ~48,000 manually segmented embryos from 1,547 worms. The model combines self-supervised masked autoencoder pretraining with supervised object detection and classification to identify embryos within the *C. elegans* uterus and classify them by developmental stage. EmbryoMAE-Det achieved high accuracy (mAP = 88.7%), with AP values of 92.8% and 84.7% for early- and late-stage embryo counts, respectively. Model-derived embryo counts showed low variability with CV%s for technical replicates below 10.4%, sufficient statistical power to detect changes as small as 5–18%, and EC_50_ values statistically indistinguishable from those obtained by manual scoring. The fully automated workflow reduces analysis time by 1,000×. In summary, this work establishes an integrated whole-organism imaging and machine-learning platform for rapid, reproducible, and high-content DART evaluation using *C. elegans* as a NAM.

## Introduction

Developmental and reproductive toxicity (DART) testing is a critical component of chemical safety assessment and has traditionally relied on mammalian models. However, increasing ethical concerns, regulatory pressure to reduce mammalian use, and the growing number of uncharacterized chemicals have accelerated the adoption of new approach methodologies (NAMs), including *in silico* models [1], *in vitro* platforms [2–4], and alternative model organisms [5–22]. Among these organisms, *Caenorhabditis elegans* has emerged as a powerful system for toxicity screening because of its genetic tractability, conserved biological pathways, and suitability for high-throughput studies [7, 9, 13, 14, 23]. The reproductive system of *C. elegans* hermaphrodites consists of two bilaterally symmetric germlines and a uterus containing embryos progressing through defined developmental stages following fertilization. This system recapitulates key biological processes relevant to human reproductive biology, including stem cell niche maintenance, cell proliferation, chromosome segregation, apoptosis, oogenesis, fertilization, and embryogenesis [24, 25]. Unlike *in vitro* systems, *C. elegans* enables toxicity assessment within an intact organism, allowing absorption, distribution, metabolism, and excretion through chemical ingestion, xenobiotic metabolism pathways, tissue-level bioavailability within multiple tissue architectures, and excretion to be evaluated in an integrated physiological context [26].

Despite these advantages, conventional *C. elegans* DART assays remain limited by labor-intensive workflows that rely on manual counting of laid embryos and hatching efficiency [27, 28]. Recent advances in flow cytometry have enabled fluorescence-based measurements of embryo defects [13]; however, these approaches are typically restricted to gross phenotypes and suffer from large variability in the data [29]. Detecting subtle changes in sub-lethal endpoints requires highly reproducible data with a low coefficient of variation (CV%). To address these challenges, we previously developed vivoDART, a high-throughput *C. elegans*-based assay that combines microfluidic immobilization using vivoChip with high-resolution imaging to quantify *in utero* embryo development [30–33]. This automated and robust immobilization protocol enables consistent alignment of ~1,000 *C. elegans* per vivoChip, facilitating high-resolution imaging and visualization of individual embryos. However, manual annotation of embryo number and developmental stage remains a major bottleneck, as it is time-consuming, requires extensive training, and is prone to subjective bias and user fatigue. Moreover, continual retraining of analysts is necessary to maintain data consistency, limiting scalability. Therefore, automating embryo detection and classification is essential for reproducible, scalable, and rapid DART assessment of uncharacterized chemicals.

Machine learning (ML) approaches have expanded the use of *C. elegans* assays for behavioral [34–36] and lifespan studies [37]. However, existing ML methods are not well suited for *in utero* embryo detection and classification because single-worm images have high spatial resolution, extreme aspect ratios, and substantial embryo overlap. In addition, fully supervised learning typically requires large number of annotated ground truth datasets, which are costly and time-consuming to generate. In contrast, recent advances in self-supervised learning, particularly masked autoencoders (MAE) [38] can learn informative feature representations from unlabeled data and have shown strong potential for microscopy image analysis [39–41]. Building on the proven effectiveness of MAE-based approaches across diverse biological imaging datasets, we apply this framework to *in utero* embryo analysis in *C. elegans*, leveraging the large volumes of high-quality, unlabeled images generated by vivoChip experiments. This approach allows the model to learn relevant morphological features directly from diverse worm images before supervised embryo detection and classification.

In this paper, we developed EmbryoMAE-Det, a 2.5D MAE-based model that combines a self-supervised pretraining network for feature extraction with a supervised object detection network for automated embryo identification and developmental-stage classification in *C. elegans*. By integrating robust feature extraction with downstream localization and classification, EmbryoMAE-Det enables consistent, scalable, and repeatable embryo phenotyping for comprehensive DART assessment. The model achieves a mean average precision of 88.7% at an intersection over union (IoU) threshold of 0.5, demonstrating accurate detection of embryos and classifying them into early- and late-stage embryos. The EmbryoMAE-Det model produced highly repeatable embryo measurements with an average CV% below 10.4% among technical replicates and detected changes as small as 18% across all embryo parameters with 80% power. When combined with body-size analysis (length, area, and volume) using our previously developed 2.5D U-Net model [31], this platform provides multiparametric DART assessment with multiple endpoints. For each chemical, full concentration-response analysis is performed across 10 concentrations and 3 biological replicates, generating data from approximately 1,200 worms. These datasets can be analyzed in ~10 minutes to provide effective concentration (*EC*_*x*_) values with narrow confidence intervals and high statistical power.

### Machine learning framework for automated *in utero* embryo phenotyping

**Figure 1.**
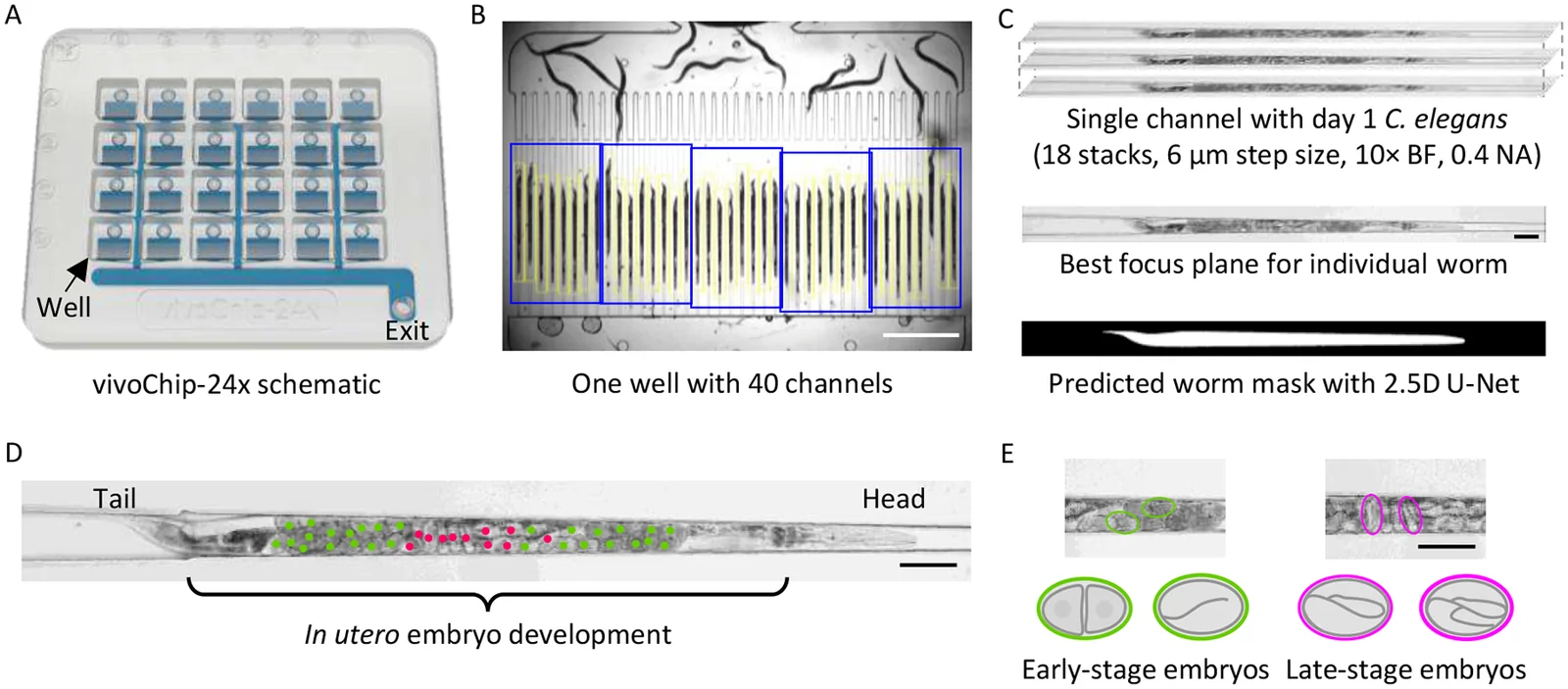
High-resolution *C. elegans* imaging using vivoChip for multi-parametric DART assay. **(A)** Schematic of the microfluidic-based vivoChip-24x device. The device contains 24 on-chip wells, each with 40 parallel microfluidic channels, to immobilize 960 *C. elegans*. **(B)** Image of 40 immobilization channels with a 2× objective. The blue boxes represent 5 imaging fields of view, optimized to capture whole body images from 8 channels per FOV with a 10× objective. **(C)** Brightfield images of *C. elegans* immobilized in individual channels were captured with 18 z-stack images using a 10× objective. An in-house machine learning model (2.5D U-Net) uses the best focal plane and ±2 adjacent planes to generate worm body masks. **(D)** Representative image of a *C. elegans* showing 39 total embryos inside the uterus. **(E)** Example images of early-stage and late-stage embryos. The schematic at the bottom shows the early-stage (green) and late-stage (magenta) embryos, with late-stage embryos referring to 2-fold or later stage embryos. Scale bars are 2 mm (B) and 100 μm (C-E).

The vivoDART assay is a high-throughput DART platform that combines microfluidic immobilization, automated brightfield imaging, and ML analysis to quantify developmental and reproductive endpoints in *C. elegans*. Unlike conventional *C. elegans* DART workflows that often require repeated manual transfers of adults, multiday brood-size analysis, and separate measurements of body size, progeny production, and hatching efficiency, vivoDART enables developmental and reproductive endpoints to be quantified from intact *C. elegans* within a single imaging-based workflow. These endpoints include developmental phenotypes such as body length, area, and volume, and *in utero* embryo phenotypes, such as embryo number and developmental stage. Our previous work demonstrated that embryo-related reproductive endpoints are more sensitive than developmental parameters and can respond independently of worm lethality for reference toxicants [30]. In vivoDART, chemical-exposed *C. elegans* are immobilized in vivoChip-24x microfluidic devices and imaged using high-resolution brightfield z-stacks to generate the data used for embryo phenotyping (see Methods, Fig. 1). A typical concentration-response study comprising 10 test conditions, 2 assay controls, and 3 independent biological replicates can generate data from as many as 1,440 *C. elegans* and more than 43,000 *in utero* embryos. Manual embryo phenotyping across such datasets is impractical and represents a significant bottleneck for large-scale chemical screening.

**Figure 2.**
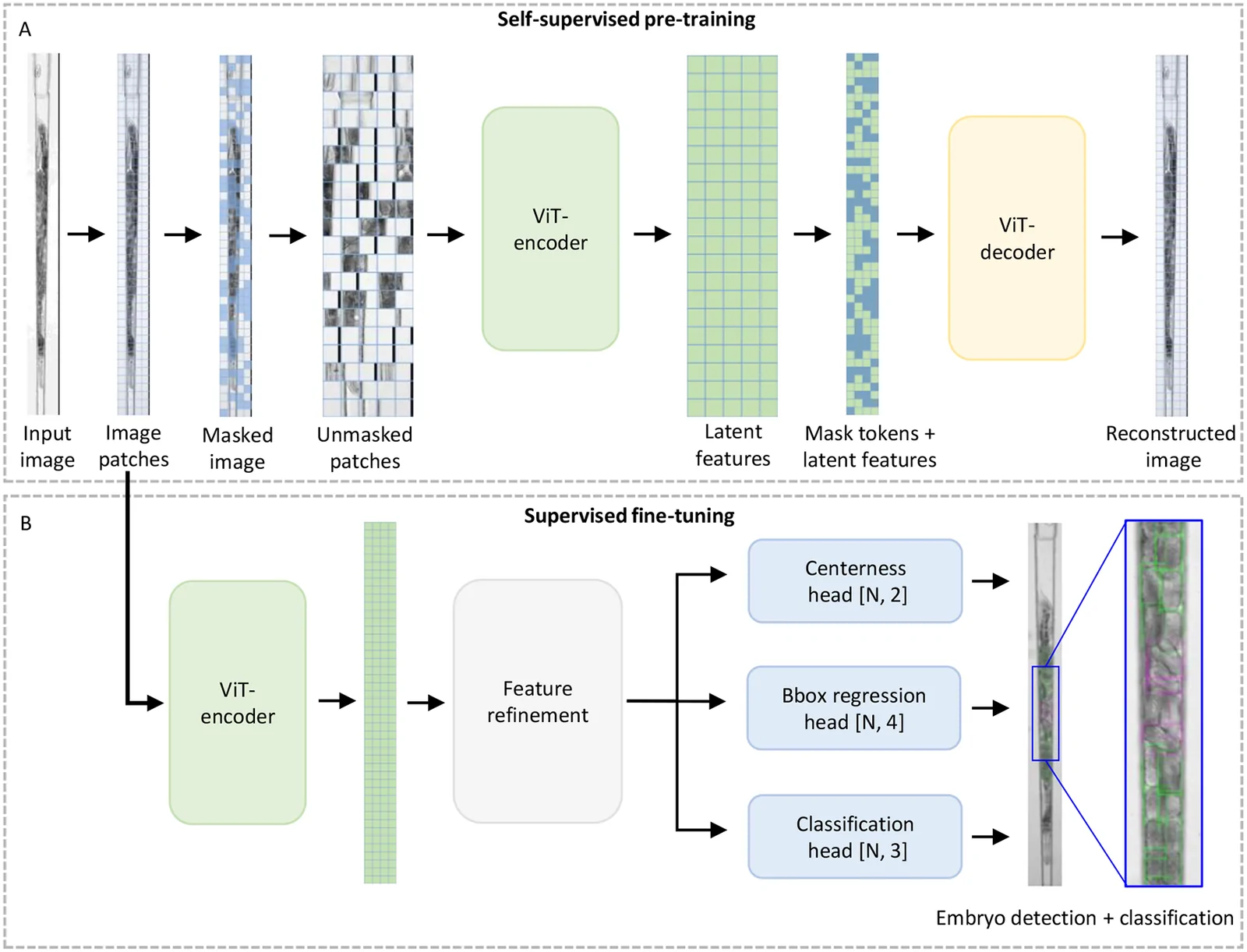
Overview of the EmbryoMAE-Det model architecture. **(A)** Self-supervised pretraining using a 2.5D masked autoencoder (MAE). The input image is divided into patches, a subset of patches is randomly masked, and the unmasked patches are encoded to obtain latent feature representations. These representations are then passed to a decoder to reconstruct the original image. **(B)** Downstream embryo detection and classification pipeline. The input image is divided into patches, which are processed by the pretrained encoder to extract feature representations. These features are fed into three prediction heads: centerness prediction, bounding box regression, and classification. Their outputs are combined to generate final embryo detections and corresponding class labels (e.g., early-stage and late-stage embryos). The zoomed-in image of the worm uterus (blue box) shows individual predicted boxes for early-stage embryos (green box) and late-stage embryos (magenta box).

To address this bottleneck, we developed EmbryoMAE-Det, a 2.5D masked autoencoder (MAE)-based framework for automated embryo detection and embryo developmental-stage classification within the vivoDART workflow. EmbryoMAE-Det uses a two-stage modeling approach (Fig. 2) to effectively leverage large-scale unlabeled imaging data while enabling accurate embryo detection and classification. In the first stage, a self-supervised MAE [38] was trained on unlabeled brightfield images of *C. elegans* in microfluidic channels to learn robust visual representations without requiring manual annotations (Fig. 2A). During this stage, a large fraction of image patches was randomly masked, and the model was trained to reconstruct the missing image content. In the second stage, the pretrained encoder was used as a feature extractor, and a supervised detection and classification network was trained using manually annotated embryo data to localize embryos within individual worms and classify them as early- or late-stage embryos (Fig. 2B).

**Figure 3.**
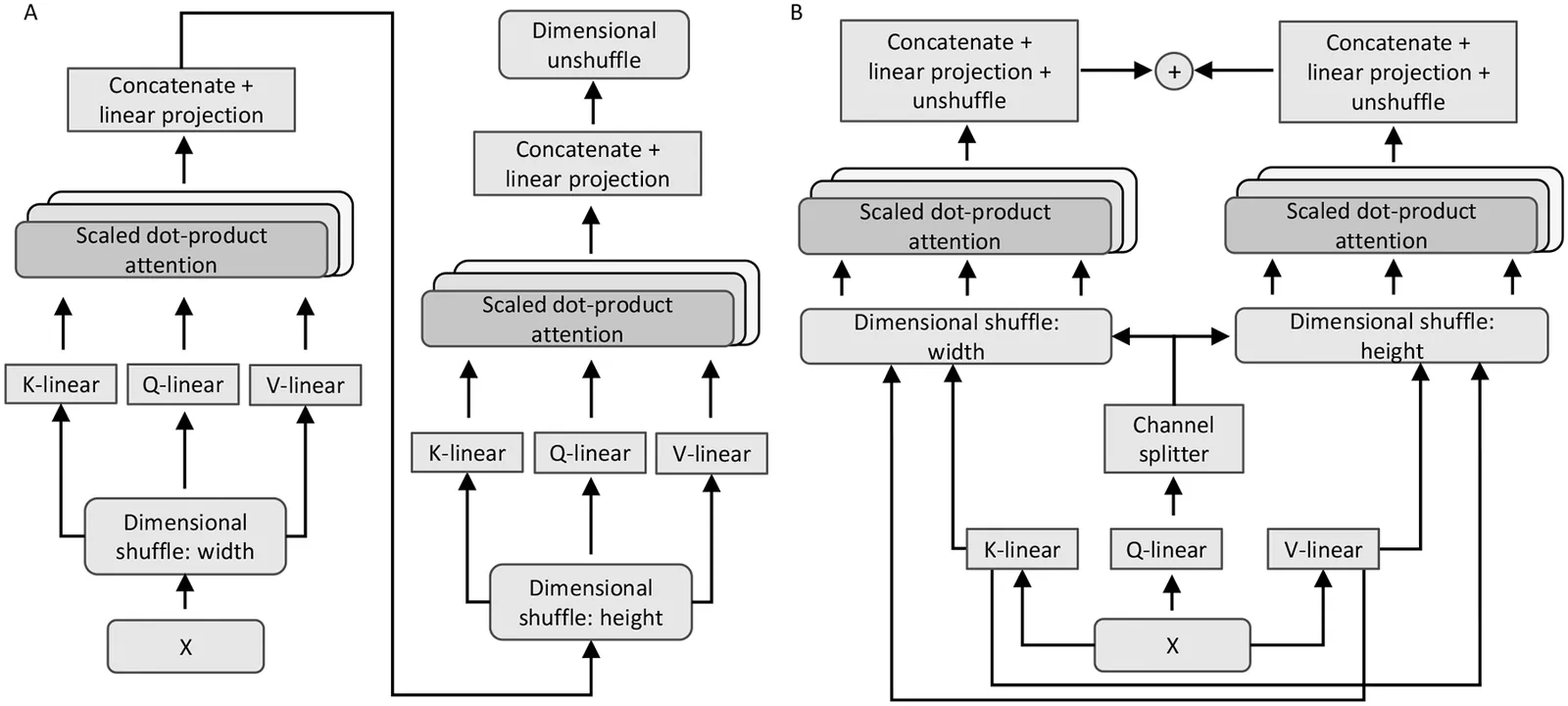
Sequential and parallel factorized attention mechanisms. **(A)** Sequential factorized attention is applied along one spatial dimension followed by the other using standard multi-head scaled dot-product attention. This enables integration of spatial information across both height and width within a single block. **(B)** Parallel factorized attention is computed independently along the height and width dimensions using shared key and value projections, while separate query projections are used for each branch. The outputs from both branches are combined to form the final representation, improving computational efficiency.

Because the input images are characterized by extreme aspect ratios (~12:1) and high spatial resolution, standard vision transformer architectures are computationally inefficient when applied directly. Therefore, EmbryoMAE-Det incorporates architectural modifications to enable efficient analysis, including convolutional tokenization and a factorized vision transformer backbone. The convolutional tokenizer embeds local spatial information into patch-level representations, while factorized attention decomposes attention along the height and width dimensions. The factorized vision transformer employs both sequential and parallel factorized attention mechanisms, applied at different stages of the network (see Methods, Fig. 3). This design reduces computational cost while preserving the ability to capture long-range spatial relationships across the full worm body.

Following self-supervised pretraining, the MAE decoder is discarded, and the pretrained encoder is used as the feature extractor for downstream embryo phenotyping. To further refine the learned representations for embryo analysis, an intermediate feature adaptation stage combines attention-based and convolutional feature refinement modules to incorporate both global contextual information and local morphological features. The resulting features are then used by task-specific heads for centerness prediction, bounding box regression, and embryo-stage classification.

During inference, each microfluidic channel is first classified as containing a full worm, partial worm, or no worm. Embryo detection is then performed only on channels containing full worms. The framework automatically identifies embryos and classifies them by developmental stage and generates three primary reproductive endpoints for each worm: the number of early-stage embryos, the number of late-stage embryos, and the total number of embryos.

The resulting framework converts high-resolution 3D brightfield images of individual *C. elegans* into quantitative reproductive phenotypes for downstream toxicity assessment. When integrated with body-size measurements and concentration-response analyses, EmbryoMAE-Det enables comprehensive and scalable DART assessment of chemicals within the vivoDART platform.

## Results

### EmbryoMAE-Det accurately detects and classifies *in utero* embryos

To develop the EmbryoMAE-Det model, we first pretrained the MAE framework using a self-supervised learning approach on 20,000 unlabeled brightfield images of microfluidic channels containing *C. elegans*. The model was then trained using the MAE-pretrained F-ViT encoder and the feature refinement modules. To assess the quality of the learned feature representations, we froze the MAE-pretrained encoder and coupled it with a lightweight decoder for supervised *C. elegans* body segmentation. This approach achieved a Dice score of 98.1%, comparable to that of a CNN-based 2.5D U-Net model (98.5%, [30]), indicating that the pretrained features effectively captured worm morphology.

To develop the final EmbryoMAE-Det model, we performed three ablation experiments to evaluate the contribution of key architectural and training components **(Supplementary Figure 1)**. First, we assessed the pretrained encoder by freezing both the encoder and feature refinement modules and training only the detection heads, thus establishing a baseline for embryo detection. Next, we enabled joint training of the feature refinement modules and detection heads, allowing task-specific adaptation of features and improving detection performance. Finally, we modified the centerness loss by combining focal loss with Dice loss to address the strong class imbalance between sparse embryo centers and background, further enhancing detection accuracy. Performance improvements between the first two configurations are further illustrated by precision-recall curves **(Supplementary Figure 2)** and average precision values (**Supplementary Table 1**). These ablation experiments informed the design of the final EmbryoMAE-Det model, with each component contributing incremental performance gains.

**Figure 4.**
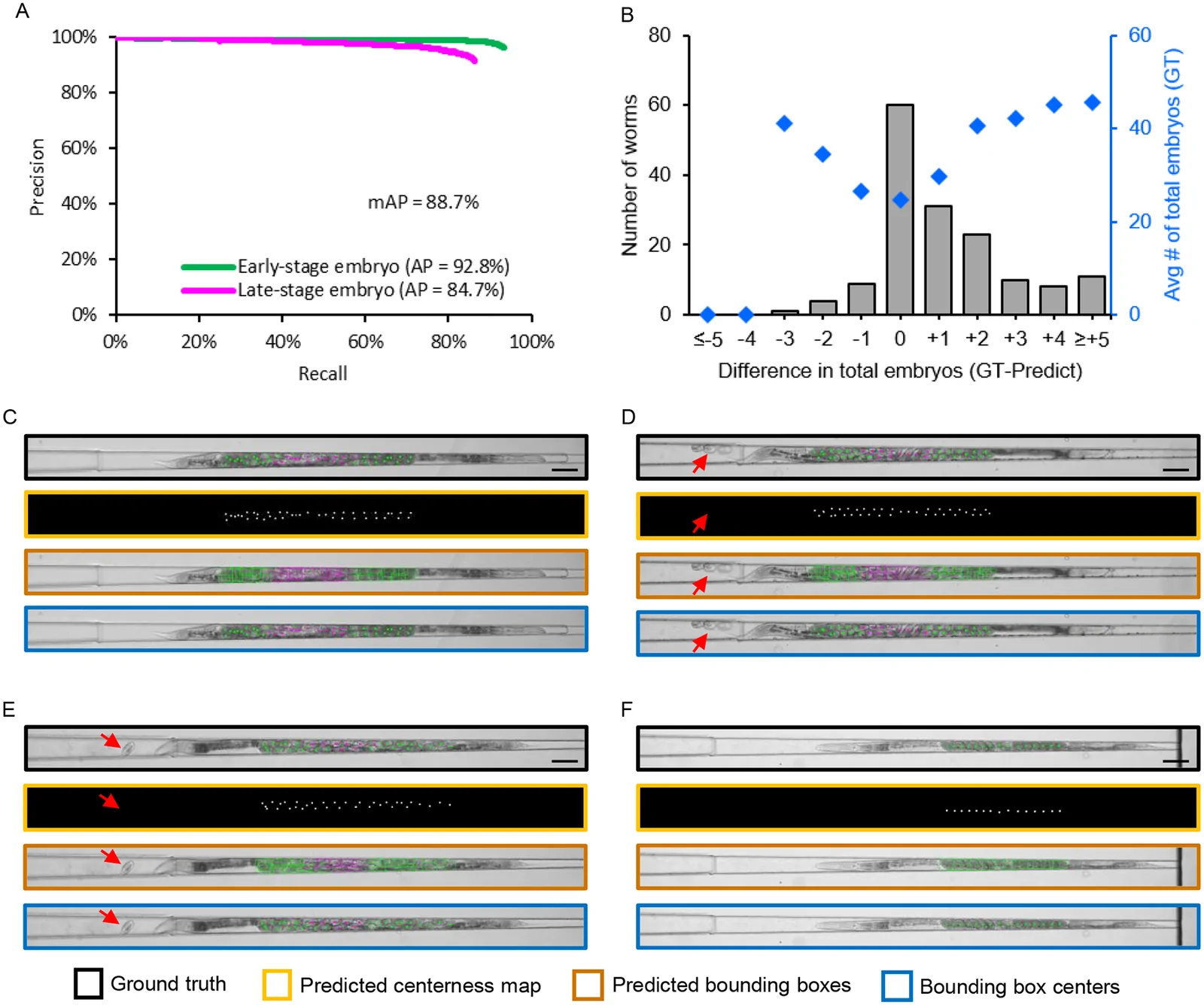
Automated analysis of *in utero* embryos using EmbryoMAE-Det model. **(A)** Precision-recall curves for early-stage and late-stage embryo detection. Average precision (AP) for each embryo class is indicated in the legend, and the overall mean average precision (mAP) is shown on the plot. **(B)** Distribution of errors in the total number of embryos across the held-out test set, calculated as the difference between manually-scored ground truth and model-predicted total number of embryos (GT - Predict; *n* = 157 channels). Gray bars indicate the number of worms in each error category, and blue diamonds indicate the average ground-truth total number of embryos per worm for channels within that error category. **(C-F)** Representative images of four test samples showing automated detection and classification of *in utero* embryos in *C. elegans*. The final EmbryoMAE-Det model identified the embryos inside the uterus and classified them as early-stage (green boxes) or late-stage (magenta boxes) embryos. For each worm, ground-truth annotations, predicted centerness maps, bounding boxes, and box centers are shown. Ground-truth embryos were manually scored and marked with green or magenta dots for early-stage and late-stage embryos, respectively. The red arrows indicate embryos located outside the *C. elegans* body, which were not predicted by the model and were not considered in the analysis. Scale bar is 200 μm.

The final EmbryoMAE-Det model was evaluated on a held-out test set of 157 channels using mean average precision at an IoU threshold of 0.5. The model achieved an overall mAP of 88.74%, with class-wise average precision values of 92.79% for early-stage embryos and 84.70% for late-stage embryos, demonstrating robust detection performance across the two embryo developmental stages (Fig. 4A). We also calculated the difference between model-predicted and ground-truth total number of embryos across the entire test set (*n* = 157). Model-predicted numbers closely matched the ground truth, with the errors centered near zero and 81% of channels falling within ±2 embryos of the manually scored numbers (Fig. 4B). The model accurately identified *in utero* embryos across worms of different sizes and successfully ignored embryos located outside the *C. elegans* body (Figs. 4C–F and **Supplementary Figure 3**). Discrepancies were primarily observed in worms with higher number of total embryos, where dense packing and overlap among *in utero* embryos made individual embryos more difficult to resolve, even for human scorers.

### ML-assisted analysis of *in utero* embryo parameters produces highly repeatable results

To validate EmbryoMAE-Det model on a larger dataset, we compared model-predicted embryo parameters with annotations generated by trained human scorers. The analyzed parameters included the number of early-stage embryos, late-stage embryos, and total embryos. The dataset was comprised of 5 experiments performed on different days, corresponding to 5 biological replicates, with 4 different DMSO concentrations (Fig. 4A). These experiments were designed to characterize assay repeatability using coefficient of variation (CV%) and assess the baseline effects of DMSO, the default solvent used in the vivoDART assay. All 960 microfluidic channels from 24 wells were analyzed in less than 5 minutes using the final EmbryoMAE-Det model. Inference outputs from all valid channels with full worms were used to calculate well averages for all three embryo parameters.

To calculate assay variability across all 5 biological replicates, we analyzed the entire dataset using the EmbryoMAE-Det model and compared the results with manually scored data (**Supplementary Figure 4**). For each DMSO condition, a total of 30 wells (5 biological replicates × 6 technical replicates) were included for each embryo parameter (**Supplementary Figures 4A-C**). The aggregated, well-level averages obtained from EmbryoMAE-Det model outputs were compared with the values obtained from manual scoring (**Supplementary Figures 4D-F** and **Supplementary Table 2**). The aggregated, well-level average numbers for the 1% DMSO condition generated by the embryo model were 30.2 ± 0.3 for early-stage embryos, 13.0 ± 0.4 for late-stage embryos, and 43.3 ± 0.4 for total embryos. Corresponding average values obtained from human scorers were 30.5 ± 0.3 for early-stage embryos (1% higher), 13.4 ± 0.5 for late-stage embryos (3.1% higher), and 43.9 ± 0.4 for total embryos (1.4% higher). Although the aggregated well-level average values were statistically different between the EmbryoMAE-Det model outputs and human scores, the absolute differences were small across all three endpoints (1.0–3.1%) supporting the practical suitability of EmbryoMAE-Det as part of a fully automated vivoDART assay.

Although automated image analysis using an ML model is substantially faster and less labor-intensive than manual analysis, a high-quality DART assay requires model performance that matches or exceeds that of expert human scorers. To characterize baseline values and variability, we calculated CV% using well averages from 6 technical replicates for each biological replicate. Across all 5 biological replicates, the average CV% values from model outputs were 4.5 ± 1.1% for early-stage embryos, 10.4 ± 1.1% for late-stage embryos, and 2.9 ± 0.4% for total embryos (**Supplementary Figure 5**). The CV% values were not significantly different between manually scored data and model outputs.

The vivoDART assay generates concentration-response data from 3 biological replicates for each chemical. To approximate a larger sampling distribution of 3-well averages, we generated combinations by selecting sets of 3 well-average values from corresponding wells across the 5 biological replicates for a given phenotype. For example, total embryos values from well A01 in 3 different experiments (exp_1_, exp_3_, and exp_5_) were combined to calculate a single 3-well average and corresponding CV% (Fig. 4B). For each well position, this process was repeated for the 10 unique combinations of 3 wells that can be generated from 5 biological replicates, across all 6 technical replicate wells. In total, 10 × 6 = 60 aggregated 3-well average and CV% values were obtained for each endpoint.

For the 1% DMSO condition, the average values across all sixty 3-well combinations were 30.2 ± 0.1 for early-stage embryos with a CV% of 5.5%, 13.0 ± 0.1 for late-stage embryos with a CV% of 15.1%, and 43.3 ± 0.1 for total embryos with a CV% of 4.7% (Figs. 4C–H, **Supplementary Figures 6A-F,** and **Supplementary Tables 3, 4**). Corresponding average CV% values from manual scoring were higher by 5.5% for early-stage embryos, 9.9% for late-stage embryos, and 17.0% for total embryos. Although the aggregated 3-well average values from model outputs differed by ≤0.6 embryos from human scores, the significant reduction in CV% values estimated from the model data was beneficial for detecting small changes in the embryo parameters.

**Figure 6.**
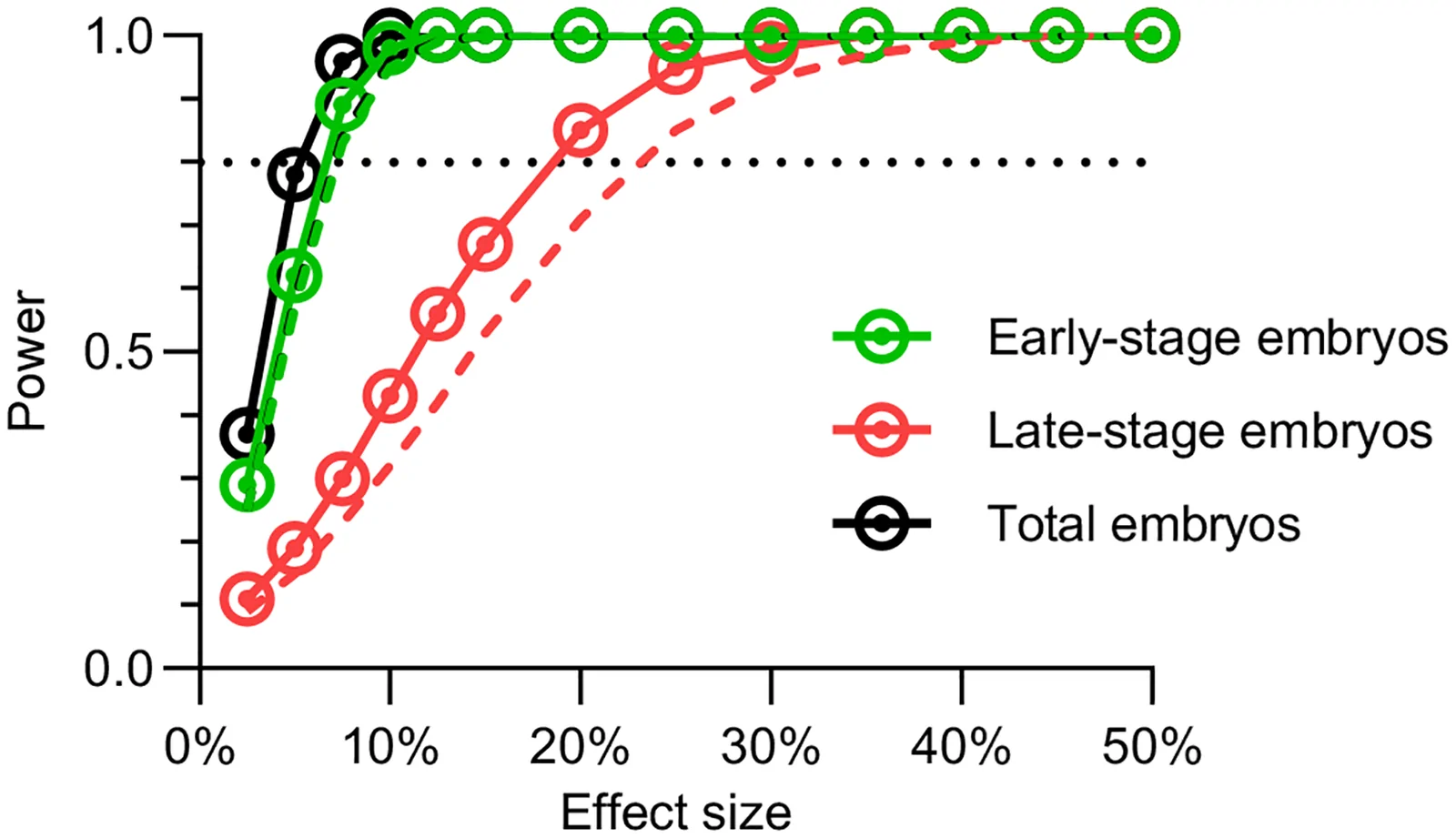
Statistical power for three reproductive parameters using 3 biological replicates. Predicted statistical power of the assay to detect hypothetical changes in early-stage, late-stage, and total embryo parameters relative to the 1% DMSO control. Power calculations used the mean and standard deviation measured from all 60 combinations of 3-replicate average values in 1% DMSO populations, with a one-sided significance level of α = 0.05. The dotted lines represent the power calculated from the manually analyzed embryo parameters. The horizontal black dotted line indicates the 80% power threshold considered acceptable for a robust assay.

We next calculated the statistical power of the assay using the data generated by the EmbryoMAE-Det model. Using the aggregated mean and standard deviation values from all sixty possible 3-well combinations in the 1% DMSO solvent control condition, we calculated the statistical power for a range of hypothetical effect sizes. The analysis indicated ≥80% power to detect changes of 6.3% in early-stage embryos, 18.3% in late-stage embryos, and 5.2% in total embryos (Fig. 6). For the same effect size, the power was higher for model-predicted data than for manually scored data. For example, the power to detect an 18.3% effect size for late-stage embryos was 67% using manually scored data, nearly 20% lower than the value estimated using model outputs. These results suggest that integrating the EmbryoMAE-Det model into the DART assay increases both the analysis speed and sensitivity for detecting small changes in sub-lethal embryo parameters, in part because of reduced variance in scoring.

### EmbryoMAE-Det accurately quantifies concentration-dependent reproductive toxicity in two reference chemicals

**Figure 7.**
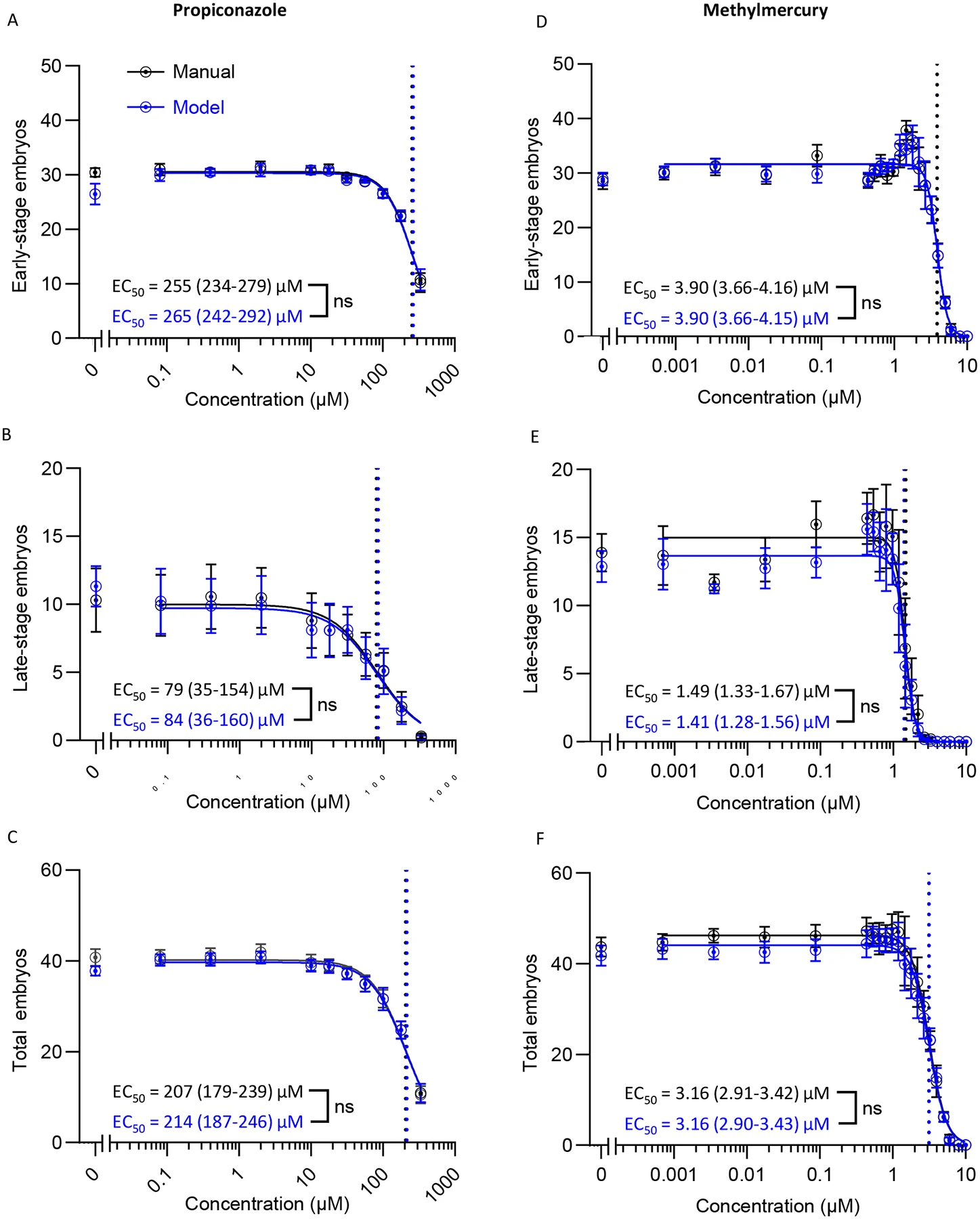
Concentration-response curves for propiconazole and methylmercury. *C. elegans* were treated with 10 concentrations of propiconazole or 20 concentrations of methylmercury for 72 hours and imaged using vivoChip devices. The brightfield images were analyzed using the EmbryoMAE-Det model (blue) and compared with manual data (black). **(A-C)** Average numbers of early-stage embryos (A), late-stage embryos (B), and total embryos (C) per worm treated with propiconazole. **(D-F)** Average numbers of early-stage embryos (D), late-stage embryos (E), and total embryos (F) per worm treated with methylmercury. Data from three biological replicates are presented as mean ± SEM. The combined well-average values were fitted with 4-parameter logistic curves for model outputs (solid blue line) and manual data (solid black line). The EC_50_ estimates are indicated with dotted lines and texts for model outputs (blue) and manual data (black). Manual and model concentration-response curves for each chemical and phenotype were compared using F-tests to determine whether the EC_50_ parameters differed significantly and *p*-values are presented as ns (not significant, *p*-value ≥0.05, see **Supplementary Tables 5,6**).

To further validate EmbryoMAE-Det model performance, we tested its ability to quantify reproductive endpoints across varying levels of induced toxicity, as required for concentration-response studies. We analyzed datasets from concentration-response experiments using two reference chemicals: the triazole fungicide, propiconazole [42–46], and the heavy metal pollutant methylmercury (II) hydroxide [11, 47–49]. The propiconazole data included 10 doses ranging from 0.08 to 334 μM, while the methylmercury data comprised 20 doses ranging from 0.0007 to 10 μM (Fig. 7). These concentration ranges were sublethal for both chemicals and were selected to examine subtle DART-specific effects.

Embryo numbers for early-stage, late-stage, and total embryos were obtained from 10× brightfield images captured using vivoChip devices and analyzed using the EmbryoMAE-Det model (**Supplementary Figure 7**). These experiments had previously been manually scored for embryo parameters using 3 biological replicates for both chemicals [30]. Concentration-response data obtained from EmbryoMAE-Det model outputs were fitted using a 4-parameter logistic curve and EC_50_ values were estimated for comparison with manually scored data. For propiconazole, model-derived EC_50_ values were 265 μM for early-stage embryos, with a 95% confidence interval (95% CI) of 242–292 μM (Fig. 7A), 84 μM for late-stage embryos, with a 95% CI of 36–160 μM (Fig. 7B), and 214 μM (for total embryos with 95% CI of 187–246 μM (Fig. 7C). For methylmercury, model-derived EC_50_ estimates were 3.90 μM for early-stage embryos, with a 95% CI of 3.66–4.15 μM (Fig. 7D), 1.41 μM for late-stage embryos, with a 95% CI of 1.28–1.56 μM (Fig. 7E), and 3.16 μM for total embryos, with a 95% CI of 2.90–3.43 μM (Fig. 7F).

The EC_50_ values from model-generated embryo numbers were not significantly different from those produced by manual scoring. For propiconazole, F-test *p*-values for EC_50_ comparisons were 0.80 (early-stage embryos), 0.98 (late-stage embryos), and 0.93 (total embryos) (**Supplementary Table 5**). For methylmercury, the corresponding F-test *p*-values were 0.99, 0.77, and 0.96, respectively (**Supplementary Table 6**).

The analysis of the complete concentration-response datasets for two chemicals involved analyzing 105 wells and took ~ 20 minutes (~0.33 hours), representing 1,000× increase in speed compared with the ~280 hours required for manual analysis (~4 minutes per worm, 150–160 minutes per well). The results presented in this study indicate that the EmbryoMAE-Det model can generate reproductive toxicity results comparable to those obtained by human scorers, while requiring only a fraction of the analysis time. When combined with our existing body model for developmental parameters (vivoBodySeg) [31], the EmbryoMAE-Det model can provide sensitive, accurate, and repeatable DART data in a high-throughput manner.

## Discussion

In this study, we present an ML-accelerated image analysis platform for automated *in utero* embryo detection and embryo developmental-stage classification in *C. elegans* to enable rapid quantification of reproductive endpoints for DART assessment. This platform was developed as part of the vivoDART assay, a high-throughput *C. elegans*-based workflow that combines microfluidic immobilization, automated brightfield imaging, and ML analysis to quantify DART endpoints [30]. To support high-content analysis of large-scale microscopy data, we developed a new embryo analysis model, EmbryoMAE-Det, utilizing a large manually annotated dataset of embryo numbers and embryo developmental stages within the worm uterus [30]. When integrated with our existing developmental toxicity analysis pipeline [31], automated embryo analysis facilitates scalable and repeatable evaluation of comprehensive DART endpoints in a whole-organism model for use as a NAM. As the number of chemicals requiring safety assessment continues to grow, automated high-throughput analysis methods are critical for efficient DART screening and for the development of safer chemicals. Additionally, rapid testing platforms, such as vivoDART, can help bridge knowledge gaps for existing, poorly characterized chemicals by providing DART-related endpoints to inform their use.

The rapid vivoDART assay leverages the vivoChip-24x microfluidic device to immobilize *C. elegans* within 960 channels across 24 wells, with each well containing a distinct worm population (40 channels per well), and to capture whole-body 3D brightfield images using a 10× objective. Automated, ML-enhanced image acquisition enables rapid and efficient imaging of every worm in the entire chip within 12 minutes, generating 2,160 images (~65 GB of data). Chronic chemical exposure, particularly at high concentrations, can result in worms with reduced body size and no *in utero* embryos. To capture a wide range of worm sizes and embryo numbers, we used two vivoChip designs: (1) vivoChip-24x-D1 for larger adult worms with >6 embryos and (2) vivoChip-24x-L4 optimized for smaller worms beyond the young L4 stage. Comprehensive DART analysis therefore required data from both devices.

The robustness of EmbryoMAE-Det was supported by a large training dataset that combined manually annotated embryo centers, developmental-stage labels, and embryo segmentations from 1,547 worm-containing channels, representing 47,852 annotated embryos. In addition, 20,000 unannotated channels were used for self-supervised learning within the MAE framework, enabling the model to learn image representations from large-scale brightfield microscopy data.

The performance of EmbryoMAE-Det highlights the value of combining self-supervised representation learning with task-specific feature refinement for embryo phenotyping in high-content *C. elegans* images across diverse experimental conditions. The pretrained F-ViT encoder provides global contextual information across the worm body. The feature refinement module combines attention and convolutional layers to capture both long-range contextual information and local morphological features during embryo detection and classification. The ablation studies further support the contribution of these design choices, showing that joint optimization of feature refinement module and detection heads, together with combined focal and Dice loss, improved overall detection performance. When combined with body classification model, this approach enables extraction of biologically relevant DART metrics and significantly reduces manual analysis time.

While the model performs robustly across experimental conditions tested here, substantial deviations in imaging conditions, worm morphology, or chemical-induced phenotypes may require model adaptation. For example, altered intestinal contrast or gut granule density in chemical-treated worms may reduce embryo visibility, impacting detection accuracy. In such cases, few-shot learning using additional representative data could improve model performance and support adaptation to new experimental conditions, as described in the literature and demonstrated for our body model [31, 50, 51].

To evaluate the performance and practical utility of the EmbryoMAE-Det model, we analyzed three datasets representing repeatability and concentration-response applications: (1) repeatability experiments with five biological replicates and six technical replicates per experiment across four DMSO solvent conditions, (2) 10-point propiconazole concentration-response assays, and (3) 20-point methylmercury concentration-response assays. Across these datasets, model outputs closely matched manual scoring, with only small absolute differences in well averages. Aggregated 3-well averages obtained from model outputs showed lower variability than manual analysis, supporting the ability of EmbryoMAE-Det to generate reproducible embryo endpoints. Model-based analysis achieved greater than 80% power to detect changes of ≥6.3% in early-stage embryos, ≥18.3% in late-stage embryos, and ≥5.2% in total embryos, whereas manual scoring could only detect a ≥23% effect size for late-stage embryos with equivalent power. In concentration-response analyses, propiconazole and methylmercury produced EC_50_ values that were not significantly different between automated and manual methods. These results demonstrate that EmbryoMAE-Det preserves the accuracy of expert manual scoring while improving throughput and reducing variability.

The integrated vivoDART platform, combining vivoChip-24x microfluidics, automated imaging, and ML-based analysis, enables considerably faster and more cost-effective DART assessment than traditional vertebrate models. The scalability of the technology together with the short lifecycle of *C. elegans* support efficient screening of large chemical libraries can be screened efficiently. When combined with existing developmental toxicity pipelines, the platform provides six DART endpoints: body length, area, and volume and the number of early-stage, late-stage, and total embryos. Among these endpoints, the number of late-stage embryos was the most sensitive endpoint for the chemicals evaluated in this study, showing significant responses at lower concentrations than traditional endpoints such as body length and lethality.

The successful adoption of NAMs depends not only on throughput but also on robustness, reproducibility, and predictive power. The high repeatability of population-level endpoints estimated from 40 worms per condition and 3 biological replicates demonstrate the robustness of the vivoDART workflow. Through integrated analysis pipelines and a searchable database, control populations can be monitored across many tests, performed over extended periods, spanning multiple thaw cycles and worm generations, and laboratory personnel. This capability provides foundation for establishing quantitative acceptance criteria, monitoring assay drift, and implementing quality control standards. Future studies will focus on evaluating concordance between *C. elegans*-based DART data and existing data from other species and humans using large panels of reference chemicals. For these large-scale studies, we plan to implement a 5-point range-finding assay, followed by 5 additional concentrations to refine concentration-response relationships within the effective range. This approach will improve EC_50_ estimations by ensuring sufficient data within the dynamic response range while maintaining the high-throughput nature of the vivoDART assay.

In summary, we developed EmbryoMAE-Det, a 2.5D MAE-based framework for automated detection and developmental-stage classification of *in utero* embryos in *C. elegans*. When integrated with existing body-analysis pipelines, this platform enables rapid, scalable, and reproducible quantification of DART endpoints. The strong agreement between automated and manual scoring, combined with improved throughput and reduced variability, demonstrates the potential of ML-assisted embryo phenotyping for large-scale chemical screening. Future work will focus on large-scale validation using diverse chemical libraries and benchmarking against established DART datasets to determine sensitivity, specificity, and translational relevance. Ultimately, this approach has the potential to advance the adoption of *C. elegans* as a practical NAM for DART assessment, reducing reliance on mammalian testing while supporting hazard identification, prioritization, and chemical safety evaluation.

## Methods

### *C. elegans* culture and chemical treatment

Wild type *C. elegans* strain N2 (Caenorhabditis Natural Diversity Resource, CaeNDR) was used for all experiments and cultured according to previously published protocols [30]. Briefly, P_0_ larval 4 (L4) stage worms grown on NGM plates seeded with HB101 *E. coli* were transferred to fresh plates and cultured for 2 days, then removed from the plates. The F_1_ generations on these plates were allowed to develop to the gravid adult stage. Gravid adults were collected and bleached using alkaline sodium hypochlorite treatment, and the resulting eggs were collected. Eggs were hatched in M9 buffer and allowed to arrest at larval 1 (L1) stage. Approximately 80 age-synchronized L1s were plated per well in 24-well culture plates with 495 μL of S media and HB101 at OD_600_ 3.0. A volume of 5 μL of chemical treatment or vehicle control was added to each well to achieve a final solvent concentration of 1.0% v/v. Plates were cultured at 20 °C for 72 hours with shaking every 6 hours to homogenize the cultures until the control population reached day 1 (D1) adulthood.

For concentration-response case studies, worms were treated with propiconazole (Cat# 45642, Sigma Aldrich, CAS# 60207-90-1, Batch# BCCD0480) or methylmercury (II) hydroxide (Cat# 13395, Alfa Aesar, CAS# 1184-57-2, Batch# M25H004). Master stocks for both chemicals were prepared by dissolving the manufacturing stock in dimethyl sulfoxide (DMSO, Cat# D2650, Sigma Aldrich, CAS# 67-68-5, Batch# RNBL9635) at 100× the highest final well concentration. Additional 100× working dilutions were prepared in DMSO for each treatment condition. Vehicle control wells received a final concentration of 1.0% DMSO. DMSO repeatability experiments were performed on 5 different days (5 biological replicates). Concentration-response data for propiconazole and methylmercury were generated using 3 biological replicates.

### High-resolution imaging of *C. elegans*

Worms were imaged 72 hours after plating and treatment using the vivoScreen high-content imaging system, as previously described [30]. Briefly, worms were transferred from the culture plate into a vivoChip-24x microfluidic imaging chip designed for *C. elegans* (Fig. 1A). Each chip contains 24 individual wells, with each well containing 40 parallel channels that taper in three dimensions. The channel geometry, combined with optimized on–off fluid pressure cycles, guides individual worms into the microfluidic channels, orients them laterally to improve visualization of internal structures, and fully immobilizes them for blur-free imaging.

Two versions of the vivoChip-24x were used depending on the developmental stage of the worms at the time of imaging. The vivoChip-24x-D1 was designed to trap D1 worms containing ≥6 embryos, while allowing laid eggs in the culture medium to pass through the microfluidic channels without clogging. The vivoChip-24x-L4 contained smaller channels optimized for efficient capture of worms up to the early L4 stage.

The vivoChip devices containing worms were imaged using a custom-designed microscope (IX73, Evident) with a large-format, scientific camera (Iris 15, Teledyne), a motorized XY stage with piezo-driven Z control (ASI), and controllable LED brightfield illumination (Sutter Instruments). Channel position (XYZ) coordinates in the chips were first mapped using an edge-detection algorithm to identify the precise locations and best focal planes of cross-shaped fiducial markers adjacent to each set of trapping channels in the microfluidic layer. Following this mapping step, automated high-resolution brightfield imaging of all channels was performed using a 10×, 0.4NA objective (UPLXAPO10X, Evident). To optimize field-of-view (FOV) positioning along the ~3 mm long channels, a low-resolution 2× image of the entire device was first captured, and a trained YOLO model was used to detect bounding boxes around each trapped worm. The positions of five 10× FOVs (2.15 × 1.26 mm^2^) were then calculated to maximize the number of full worm bodies captured, which were staggered across the 40 channels (Fig. 1B). To capture entire worm bodies and all *in utero* embryos, z-stack images consisting of 18 slices were acquired with 6 μm step sizes. Each vivoChip-24x device generated 2,184 TIFF images, corresponding to ~65 GB of data.

### Pre-processing of images

All raw 16-bit TIFF images and experimental metadata were transferred from the imaging system to an in-house, 500 TB data server. The images were first converted to Zarr format using the known trapping channel positions derived from the fiducial-marker detection during chip mapping. This file structure enabled block-wise read access to individual channels by spatially slicing the imaging data (Fig. 1C). This pre-processing step reduced disk read times while accessing relevant image data, such as during body-mask prediction using the 2.5D U-Net model (Fig. 1C, [31]).

### Manual scoring, classification, and segmentation of *in utero* embryos

Ground-truth annotations of *in utero* embryos from individual *C. elegans* were generated using two in-house graphical user interface (GUI) tools: vivoAnalyzer (**Supplementary Figures 8, 9**) for embryo scoring and classification, and vivoSegmenter (**Supplementary Figures 10, 11**) for embryo segmentation. The vivoAnalyzer allowed users to open images of immobilized *C. elegans* acquired from the vivoChip device and scroll through z-slices for each cropped channel. Using our 2.5D U-Net model, each channel was classified into one of three classes: full worm (entire worm body present inside the channel), partial worm (partially visible worm inside the channel), or no worm (empty channel). Only channels classified as full worm were used for manual scoring, classification, and segmentation of *in utero* embryos.

Manual embryo scoring, performed in vivoAnalyzer, was defined as the process of quantifying the number of *in utero* embryos within each immobilized *C. elegans* and followed by assigning each embryo to a developmental stage. Users opened individual channel images classified as full worm and scored all visible *in utero* embryos within the *C. elegans* (Fig. 1D). This task was performed by placing a dot at the center of each visible embryo and assigning the developmental stage as either early-stage or late-stage embryo, based on the 2-fold embryo development as the classification threshold (Fig. 1E).

Manual embryo segmentation was defined as the process of delineating each *in utero* embryo within a *C. elegans* to clearly distinguish its boundaries from those of adjacent embryos. Individual channels containing immobilized worms that had been previously scored with embryo center dots and class labels were opened in vivoSegmenter. Users selected one embryo at a time and clicked along the embryo edges, encompassing the entire embryo in a closed polygon. The GUI recorded the spatial coordinates (X, Y, and Z) of each point clicked by the user. After all points for an embryo were entered and connected to form a closed shape, a polygon was generated representing the embryo boundary. Because embryos can span across multiple z-planes, accurate segmentation typically required examining ±2 z-slices around the best focal plane. All scoring and segmentation data were stored in the database. During model training, class labels and segmentation annotations were retrieved, and each segmentation was converted into a bounding box representation for training the embryo detection and classification model.

### Data pre-processing for ML analysis

For each channel, 18 z-stack images were acquired, with most embryos located within 5 z-slices around the best focal plane. The best focal plane for each worm was identified by applying Laplace transform to each z-slice and selecting the slice with the highest frequency components [52, 53]. To incorporate local depth information, a total of 2*N* + 1 slices (*N* = 2) centered around the best focal plane were selected and stacked along the channel dimension, forming a 2.5D representation of size (2*N* + 1) × *H* × *W*. Following slice selection, all images were standardized in size. The image height was fixed at 5,056 pixels, while the image width, which varied across channels, was symmetrically padded to a fixed width of 368 pixels, resulting in images of 5,056 × 368 × 5 pixels. Finally, the image axes were transposed such that the spatial dimensions were reordered to 5 × 5,056 × 368 to ensure consistent orientation for downstream processing.

### Detailed EmbryoMAE-Det model architecture

EmbryoMAE-Det was implemented as a 2.5D masked autoencoder-based embryo detection and classification framework. Raw input images were converted into patch embeddings using a convolutional tokenizer with a patch size of 8 × 8, replacing the standard linear projection. The tokenizer consisted of convolutional layers with residual connections, which introduced local inductive biases and embedded spatial context directly into the token representations. This design eliminated the need for explicit positional embeddings in the encoder and improved training stability [54–56]. Consistent with previous research on MAE for images, 75% of the tokenized input was randomly masked during pretraining, reducing computational costs.

The encoder was implemented as a factorized vision transformer (F-ViT) with 16 layers and an embedding dimension of 512. The encoder operated exclusively on visible tokens and was designed to efficiently process high-resolution inputs. Because the patch size was reduced from the standard 16 × 16 to 8 × 8, we used a slightly smaller encoder feature dimension than the canonical ViT, 512 rather than 768. The decoder was implemented as a lightweight transformer with 8 layers and an embedding dimension of 160. This value was chosen to approximate the 3:1 ratio between encoder and decoder embedding dimensions used in the original MAE framework. The decoder received the encoded visible tokens along with learnable mask tokens corresponding to the masked patches as inputs. Fixed sinusoidal positional embeddings were added to the decoder input to provide spatial information [57]. The decoder employed cross-attention, where mask tokens attended to the encoded representations to reconstruct the missing patches **(Supplementary Figure 12)**.

High-resolution microscopy images with extreme aspect ratios (5,056 × 368 pixels) present a significant challenge for standard vision transformer architectures because the computational cost of global self-attention scale quadratically with the number of tokens [58]. For an image size of *H* × *W*, the number of tokens scales with *HW*, making full self-attention computationally expensive.

To address this challenge, we employed F-ViT backbone that decomposes attention along the spatial dimensions, inspired by efficient attention mechanisms used in video modeling [59]. The input image $X\in {\mathbb{R}}^{H\times W\times C}$ was tokenized and reshaped into a sequence representation $Z^{\left(0\right)}\in {\mathbb{R}}^{N_{h}\times N_{w}\times D}$, where *N*_*h*_ and *N*_*w*_ denote the number of patches along the height and width dimensions, respectively, and *D* is the embedding dimension.

Instead of computing full 2D self-attention, attention was factorized separately along the height and width dimensions. This factorization substantially reduced computational complexity while preserving the ability to capture long-range dependencies across both spatial axes. In the initial layers, we used sequential factorized attention (Fig. 3A), where attention was first computed along one spatial dimension followed by the other. This enabled efficient propagation of information across the full image extent. In deeper layers, we used parallel factorized attention (Fig. 3B), where attention was computed independently along the height and width dimensions and the resulting representations were combined.

This design improved computational efficiency while maintaining expressive feature interactions. Formally, given an input tensor *Z*^(*l*−1)^, attention along the height and width dimensions was computed separately, and the outputs were aggregated as:

(1) $$A_{hw}=\text{Proj}\left({\text{Attn}}_{\text{height}}\left(Q_{h},K_{h},V_{h}\right)+{\text{Attn}}_{\text{width}}\left(Q_{w},K_{w},V_{w}\right)\right),$$

where Attn_height_ and Attn_width_ denote self-attention computed along the respective spatial axes.

Each attention block was followed by feed-forward network implemented as gated linear unit with GeLU activation and an expansion factor of 4×, which improved model capacity and training efficiency [60].

### Self-supervised network pretraining using unlabeled *C. elegans* images

The F-ViT model was pretrained using a self-supervised reconstruction objective on a large corpus of unlabeled *C. elegans* imaging data. The dataset comprised 20,000 microfluidic channels collected across 27 independent experiments. During pretraining, a large fraction of input patches was randomly masked, and the model was trained to reconstruct the missing regions by minimizing the mean absolute error loss between reconstructed and original pixel intensities. The loss was computed only over the masked tokens, following the MAE training paradigm. Pretraining was performed for 500 epochs using the AdamW optimizer, with a base learning rate of 0.001 and a weight decay of 0.01. To accommodate hardware memory constraints while maintaining a large effective batch size, gradient accumulation was employed. Training was performed using FP16 mixed precision to improve computational efficiency and reduce memory usage. To enhance robustness against variations in imaging conditions, data augmentation was applied during pretraining, including random rotations, horizontal and vertical flips, and contrast adjustments.

### Supervised adaptation model for embryo detection and classification

Following self-supervised pretraining, the MAE decoder was discarded and the pretrained F-ViT encoder was used for downstream embryo detection and classification. Rather than directly applying detection heads to the pretrained features, we introduced an intermediate supervised feature adaptation stage to bridge the gap between generic representations and task-specific requirements. In this stage, the pretrained tokenizer and encoder were retained, and additional feature refinement modules were introduced to capture both global and local spatial information. These modules consisted of complementary attention-based and convolutional branches applied to the encoder outputs. The outputs from these branches were concatenated to form a fused feature representation. To guide this adaptation, a lightweight decoder was attached to perform worm body segmentation, providing dense supervision that encouraged the model to capture biologically meaningful morphology and spatial context. After training, the segmentation decoder was discarded, while the tokenizer, encoder, and feature refinement modules were retained. The final detection model was then constructed by attaching task-specific heads for embryo centerness prediction, bounding box regression, and embryo developmental stage classification. This design enabled the model to leverage both the global representations learned during self-supervised pretraining and the spatially enriched features obtained through the intermediate refinement stage.

The embryo detection model was built upon the feature representations produced by the intermediate refinement stage and followed a center-based, anchor-free regression paradigm [61]. The network predicted embryo locations using a centerness map, followed by bounding box regression and classification. A localization head, implemented as a U-Net style decoder with transposed convolutions, produced a 2D centerness heatmap in which each spatial location represented the likelihood of an embryo center. This heatmap was used to identify candidate regions for bounding box regression. Bounding box regression was performed at these candidate locations by predicting offsets relative to the candidate location along with the corresponding bounding box dimensions (height and width). This formulation enabled direct localization without the use of predefined anchor boxes. To determine the developmental stage of each detected embryo, region-of-interest features were extracted from the predicted bounding boxes and passed through a multi-layer perceptron for classification into early-stage embryo, late-stage embryo, or background classes [62].

### Supervised network training using labeled *C. elegans* images

We evaluated the impact of feature adaptation and loss design through three ablation experiments. All variants shared the same architecture, including the pretrained encoder, feature refinement modules, and detection heads, but differed in parameter-training strategy and centerness supervision. Specifically, we evaluated: (i) frozen features, where only the detection heads were trained (experiment 1); (ii) joint training, where the feature refinement modules and detection heads were optimized together (experiment 2); and (iii) joint training with a modified centerness loss, where focal loss was combined with Dice loss to address class imbalance. This third configuration was implemented in the final model (EmbryoMAE-Det). Unless otherwise specified, complete intersection over union (CIoU) loss and focal loss were used for bounding box regression and classification, respectively. These configurations allowed us to examine how different training choices influenced the detection model performance.

The detection model was trained in a supervised manner using the pretrained F-ViT encoder and the feature-refinement modules from the intermediate refinement stage. During training, the encoder weights were kept fixed, while the feature refinement modules and detection heads were optimized jointly. Training was performed for 1,500 epochs using the AdamW optimizer with a learning rate of 5 × 10^−4^ and a weight decay of 1 × 10^−4^. Gradient accumulation was used to enable large effective batch sizes, and FP16 mixed-precision training was used to improve computational efficiency. To reduce overfitting, dropout was applied to both pretrained and newly initialized layers, with higher dropout rates used for the newly initialized layers.

The dataset consisted of 1,547 channels with a total number of 47,852 embryos that were manually scored and segmented (**Supplementary Figure 13**). The entire dataset was divided into training, validation, and test sets using an 8:1:1 split (1,237 training, 153 validation, and 157 testing samples). To improve robustness and enable analysis of data captured under varying imaging conditions, data augmentation was applied during training, including random rotations, horizontal and vertical flips, and contrast adjustments. A multi-task loss function was used to jointly optimize localization and classification objectives. The total loss was defined as a weighted sum of three components: centerness prediction, bounding box regression, and classification. The centerness loss combined focal and Dice losses to address class imbalance arising from the dominance of background regions. Bounding box regression was optimized using the CIoU loss, which accounts for overlap, center distance, and aspect ratio consistency between predicted and ground-truth boxes. Classification was supervised using focal loss to handle class imbalance across embryo stages. All the models were trained on a workstation with 128 GB RAM and two NVIDIA A6000 GPUs, each with 48 GB VRAM.

### Model evaluation metrics

Model performance was evaluated using task-specific metrics for embryo detection and classification, body classification, and body segmentation. Embryo detection and classification performance was assessed using mean average precision at an IoU threshold of 0.5, following standard object-detection evaluation protocols [63]. A predicted bounding box was considered a true positive if its intersection-over-union (IoU) with a ground truth box exceeded 0.5 and the predicted class label was correct. The IoU was defined as:

(2) $$\text{IoU}=\frac{\left|B_{p}\cap B_{g}\right|}{\left|B_{p}\cup B_{g}\right|},$$

where *B*_*p*_ and *B*_*g*_ denote the predicted and ground truth bounding boxes, respectively. For each class, precision-recall curves were computed by varying the detection confidence threshold, and the average precision (AP) was calculated as the area under the precision-recall curve. The final mean average precision (mAP) score was computed as:

(3) $$\text{mAP}=\frac{1}{C}\overset{C}{\underset{c=1}{\sum }}AP_{c},$$

where *C* denotes the number of classes.

For body classification, performance was quantified using the weighted F1 score, which balances precision and recall while accounting for class imbalance. The F1 score was defined as:

(4) $$F1=2\cdot \frac{\text{Precision}\cdot \text{Recall}}{\text{Precision}+\text{Recall}},$$

and the weighted variant was computed as the class-frequency weighted average across all classes.

For body segmentation, accuracy was evaluated using the Dice score, a standard metric for segmentation tasks, which measures the spatial overlap between predicted segmentation masks and ground truth masks. The Dice score was defined as

(5) $$\text{Dice score}=\frac{2\;\left|A\cap B\right|}{\left|A\right|+\left|B\right|},$$

where *A* and *B* denote the sets of predicted and ground truth pixels, respectively. The Dice score ranges from 0 (no overlap) to 1 (perfect overlap), reflecting segmentation quality.

### Post-processing and inference for extracting *C. elegans* embryo parameters

During inference, embryo-related parameters were computed by integrating predictions from the body classification and embryo detection models (**Supplementary Figure 14**). First, each channel was processed using the body classification model, which assigned one of three classes: full worm, partial worm, or no worm. For channels classified as full worm, the embryo detection model predicted bounding boxes and corresponding class labels (early-stage embryos, late-stage embryos, and background). To refine the detections, non-maximum suppression (NMS) was applied with an IoU threshold of 0.75 to remove redundant overlapping predictions. Three embryo numbers were computed by aggregating the detections per class, yielding the number of early-stage and late-stage embryos as well as the number of total embryos computed as the sum of early- and late-stage embryo detections.

The body classification and segmentation models used for inference were adapted from the previously proposed vivoBodySeg framework [31]. In contrast to the original architecture, which jointly learned classification and segmentation using a shared encoder, the two tasks were decoupled into separate models to enable independent optimization [30]. Specifically, the classification model consisted of a convolutional encoder followed by a ViT bottleneck and a classification head, and operated on a single z-slice, reflecting its reliance on global morphological features. In contrast, the segmentation model used a 2.5D architecture with a convolutional encoder, a ViT-based bottleneck, and a convolutional decoder. The segmentation model was trained using five z-slices stacked along the channel dimension to incorporate volumetric context.

### Statistical analysis of concentration-dependent embryo-related parameters

Model outputs for both body sizes (length, area, and volume) and embryo numbers (number of early-stage, late-stage, and total embryos) were first cleaned to remove invalid samples. Specifically, we excluded empty channels, partial worms in which part of the body mask extended outside the FOV or protruded more than 200 μm from the trapping channel exit. Additionally, channels where skeletonization failed, preventing reliable body-length estimation, were excluded. Tukey filtering was used to identify biological outliers. Worms with body volume or total number of embryos exceeding 1.5× the interquartile range from the well-population median were excluded. Cleaned and filtered well averages were used to generate plots for the different embryo parameters.

Assay quality was evaluated using the CV% and statistical power. Inter-experimental variability was quantified using CV% (CV = σ/μ). The aggregated means and standard deviations were derived from all possible combinations of three 1% DMSO wells within the repeatability experiments (6 technical replicate wells, repeated over 5 different biological replicates). Assay power was calculated using hypothetical effect sizes ranging from 2.5% to 50% relative to the aggregated control mean and standard deviation. Power calculations were performed using a one-sample, one-tailed *t*-test (α = 0.05) in GraphPad Prism. The well-level and aggregated mean values for all three embryo parameters were assessed for normality using the Kolmogorov–Smirnov test. Because the majority of the datasets did not follow a normal distribution, differences between model-derived outputs and human annotations were evaluated using the Wilcoxon matched-pairs signed-rank test.

Concentration-response curves were generated using a 4-parameter logistic curve model (“Find ECanything” nonlinear fit function in GraphPad Prism 10.6.1). For all three embryo endpoints (early-stage, late-stage, and total embryos), the lower asymptote parameter was constrained to zero, as L4 worms are expected to carry no embryos. The 95% asymmetric confidence intervals were reported for each EC_50_ estimate. Two concentration-response curves were compared using an F-test to determine whether the best fit EC_50_ values and Hill slope values differed between the two datasets.

## Supplementary Files

This is a list of supplementary files associated with this preprint. Click to download.

- EmbryoModelPaperSupFINAL.pdf

## Figures and Tables

**Figure 5.**
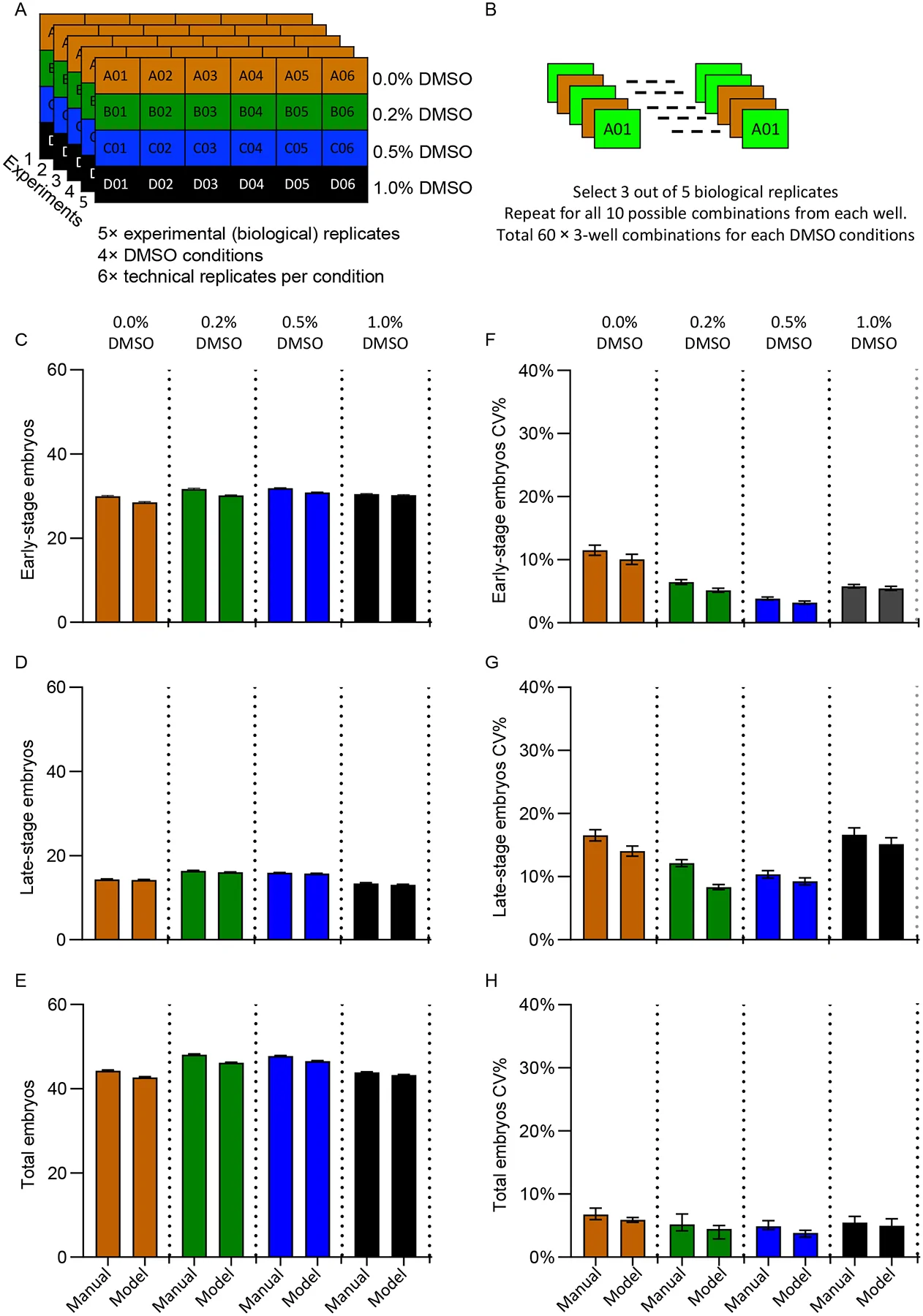
Automated embryo analysis using EmbryoMAE-Det model compared with manually scored ground truth. **(A)** Plate map showing 4 DMSO treatment conditions (0.0%, 0.2%, 0.5%, and 1.0%). The same plate map was used for 5 biological replicates, performed on different days. **(B)** Schematic, illustrating all sixty 3-well combinations from 5 experiments and 6 technical replicates for each DMSO condition. **(C-E)** Bar plots of aggregated averages from all sixty 3-well combinations for early-stage embryos (C), late-stage embryos (D), and total embryos (E) across the 4 DMSO conditions. **(F-H)** Bar plots of average CV% values from all sixty 3-well combinations for early-stage embryos (F), late-stage embryos (G), and total embryos (H) for 4 DMSO conditions. Average well values obtained from EmbryoMAE-Det outputs (Model) and the manual scoring (Manual) are presented. The raw data are presented in **Supplementary Figure 6**. The pair-wise comparison was performed, and *p*-values are presented in **Supplementary Tables 3, 4**.

## Data Availability

The datasets generated and/or analyzed during the current study are available from the corresponding authors upon reasonable request.
